# The value of eosinophil count in the diagnosis of preeclampsia among pregnant women attending the University of Gondar Comprehensive Specialized Hospital, Northwest Ethiopia, 2021

**DOI:** 10.1186/s12884-022-04892-9

**Published:** 2022-07-12

**Authors:** Yemataw Gelaw, Fikir Asrie, Muluken Walle, Zegeye Getaneh

**Affiliations:** 1 College of Medicine and Health Sciences, School Biomedical and Laboratory Sciences, Department of Hematology and Immunohematology, University of Gondar, Gondar, Ethiopia; 2 College of Medicine and Health Sciences, Department of Medical Laboratory Science, Jigijiga University, Jigijiga, Ethiopia

**Keywords:** Clinical significance, Eosinophil count, Ethiopia, Preeclampsia, Pregnancy

## Abstract

**Background:**

Currently, studies showed that eosinophil count had clinical significance in the diagnosis and prognosis of diseases. But, the clinical significance of eosinophil count in pregnancy specifically in preeclampsia (PE) is not well studied. The main objective of the present study was to assess the diagnosis value of eosinophils counts among pregnant women with PE.

**Methods:**

A comparative cross-sectional study was conducted on a total of 126 pregnant women at the University of Gondar Comprehensive Specialized Hospital, using a convenient sampling technique. Socio-demographic and clinical data were collected by questionnaire and datasheet from patient’s charts, respectively. A total of six ml of blood was collected from each study participant; three ml for complete cell count analysis using Sysmex XS-500i hematology analyzer and three ml for liver function tests using Humastar 200 chemistry analyzer. The data were entered into Epi-data and exported to SPSS 20 for analysis. The independent t-test was used for normally distributed data and, the Mann–Whitney U test was used for non-normally distributed data. Binary logistic regression and receiver operative curve analyses were also done to assess the diagnosis value of eosinophils count. *P*-value < 0.05 was considered statistically significant.

**Results:**

The eosinophils count of PE pregnant women were significantly lower than the normotensive (NT) pregnant women (median (IQR): 50 (10—200) vs. 120 (60 – 270); (*p* = 0.002). The eosinophil count ≤ 55 cells/µL had an AUC of 0.66 (95% CI; 0.56—0.75) for diagnosis of PE with a sensitivity of 50.8%, specificity of 77.8%, and positive and negative predictive value of 69.6% and 61.3%, respectively. The abnormal AST and ALT results were significantly higher among PE pregnant women compared to NT pregnant women (AOR: 14.86; 95% CI: 4.97—44.4 and Fischer exact test *p*-value = 0.001, respectively).

**Conclusion:**

The eosinophil count ≤ 55cells/µl had a reasonable/acceptable AUC which may use in the diagnosis of PE. AST and ALT were also significantly higher in PE pregnant women compared to NT pregnant women. Multicenter longitudinal studies with a large sample size are recommended to verify the role of eosinophil count in the diagnosis of PE.

## Background

Pregnancy-related complications, such as gestational diabetes mellitus and preeclampsia (PE) are potent contributors to mortality and morbidity in pregnant women. Early diagnosis and prediction of these complications are crucial to improving their outcomes [1]. PE is defined as a new onset of hypertension associated with proteinuria (blood pressure:140 / 90 mmHg after 20^th^ GW; proteinuria: ≥ 1 + in urine dipstick) and fluid retention detected for the first time after the 20th week of gestation and affects 2–8% of all pregnancies [2, 3]. It can be classified as early-onset PE when PE was diagnosed before 34 weeks of gestation, late-onset PE when diagnosed after 34 weeks of gestation [4].

The maternal immune system is strongly stressed during all stages of gestation. It is known that normal pregnancy is accompanied by leukocytosis and the most cause is an increase in neutrophils. Leukocytosis is also significant in PE [5]. Studies have shown that the hematological parameters in pregnant women with hypertensive disorder pregnancy, including PE, are different from Normotensive (NT) pregnant women- (pregnant women with normal blood pressure; less than 120/80 mmHg and negative for urine protein [6]) [7]. Specifically, neutrophil to lymphocyte ratios (NLR) and platelet count with their indices may predict disease development and may help in monitoring disease and the prognosis of PE [1, 3, 8–13]. However, differential counts of leukocytes (neutrophils, lymphocytes, monocytes, basophils, and eosinophils) in patients with PE have not been defined precisely [5].

Eosinophils are granular nucleated white cells representing up to 6% of the bone marrow cells and are routinely measured as part of the complete blood cell count. Their development and maturation occur in the bone marrow underexposure of myeloid precursors to interleukin-3, Granulocyte–macrophage colony-stimulating factor, and interleukin-5 (IL5). IL5 is particularly important for the final stage of eosinophil differentiation and migration into the circulating blood. Furthermore, it is a key cytokine in the survival of circulating and tissue eosinophils. It prevents eosinophils from apoptosis and promotes cell activation [14]. The activation of eosinophils usually occurs after they have migrated into a tissue site by the integrated interactions of cytokines, chemokines and adhesion molecules [15].

Eosinophils play an important role in the immune response to infection including parasites and fungi [16]. They are also known to play important roles in the pathogenesis of allergic inflammation by secreting various mediators like eosinophil cationic protein, eosinophil derived neurotoxin and major basic protein upon activation by cytokines, immunoglobulins, or platelet-activating factors [15]. They are also important in tissue development, repair, support, and maintenance of tissue integrity [16].

Eosinophilia is a common hematological term and is defined as an increased eosinophil count above the normal range in the peripheral blood. Even though different articles define eosinophilia at different cut off values, most study articles were used a cut off value > 450 cells/µL to define eosinophilia [17–19] and always associated with infection (parasites and fungi), allergic inflammation and chronic inflammatory [17, 18, 20, 21]. On the other hand, eosinopenia is the term used to describe a low eosinophil count. However, since the normal reference range of eosinophil includes zero, most of the time it is theoretical and has no clinical significance [16]. Nevertheless, currently, studies showed that eosinopenia had clinical significance in the diagnosis and prognosis of diseases [8, 22–24]. The problem is the cut-off value of eosinophil count to define eosinopenia and the study articles used a different cut-off value of eosinophil count [16, 22, 24].

Preeclampsia affects the gene expression, production and secretion of different molecules in the body which are crucial for the regulation of eosinophils in the peripheral circulation. It increases type 1 interferons that induced eosinophil cell apoptosis and decrease interleukin-5 which is important for differentiation and survival of circulating Eosinophils [14, 22, 25, 26]. Preeclampsia can also cause stress in pregnant women which is one of the main causes of eosinopenia [27, 28]. These all possible reasons may reduce the circulating eosinophil count and can be an indicator of the presence of preeclampsia in pregnant women. But, the clinical significance of eosinophil count in pregnancy specifically in PE is not well studied. Additionally, the identification of sensitive specific, cost-effective, and simple-to-use biomarkers for the diagnosis of PE is a critical goal in modern obstetrics. The presence of PE can be early estimated by using Doppler ultrasound [29, 30]. Doppler ultrasound is a non-invasive technique for evaluating uteroplacental circulation, but it is not recommended for regular screening of PE [31, 32]. Moreover, it requires proper sonographer training and adherence to a standard ultrasound methodology to establish uniformity of results among different operators, and it is not readily available in many hospitals and health care centers in developing countries [30]. Furthermore, this test has a high false-positive rate, which may lead to excessive patient anxiety and increased healthcare costs [33]. Therefore, the main purpose of the present study was to assess the clinical significance of eosinophils counts in patients with PE and compare them to those in normal pregnancy. In the current study, Eosinophilia was defined as eosinophil count > 450 cells/µL [19] and eosinopenia was defined as eosinophil count ≤ 55 cells/µL which had a better combination of sensitivity and specificity in the diagnosis of PE.

## Materials and methods

### Study area

The study was conducted at the University of Gondar Comprehensive Specialized Hospital Antenatal care (ANC) unit. The hospital is located in Gondar town, Central Gondar Zone, Amhara regional state, Ethiopia. Gondar town is located 738 km away from Addis Ababa, the capital city of Ethiopia and 175 km far from Bahir Dar city, the capital city of Amhara regional state in the northwest direction (Central Gondar Zone Road and Transport Administrative). The town is situated at a latitude and longitude of 12°36’N 37°28’E with an elevation of 2133 m above sea level [34]. Currently, the hospital has been serving people from Central, North and Western Gondar Zone as well as the surrounding district’s region. The hospital provides medical services, including internal medicine, pediatrics, surgery, gynecology/obstetrics, psychiatry, ophthalmology, and maternal and child care. ANC clinic is one of the units under the department of gynecology and obstetrics. The clinic serves around 17,000 pregnant women per year. Currently, there are 16 gynecologists and 27 full times midwives working at the University of Gondar Comprehensive Specialized Hospital ANC unit.

### Study design and period

A hospital-based comparative cross-sectional study was conducted to evaluate the clinical significance of Eosinophils as potential markers for the prediction of PE among pregnant women attending the University of Gondar Comprehensive Specialized Hospital ANC unit, Gondar, Northwest Ethiopia from March 9, 2021, to May 13, 2021.

### Source and study population

All pregnant women who visited the ANC unit at the University of Gondar Comprehensive Specialized Hospital were the source populations and pregnant women with PE after the 20^th^ gestational week (GW) who attended the University of Gondar Comprehensive Specialized Hospital ANC unit during the study period were taken as the study population for the case group. Whereas, age and GW matched NT pregnant women seeking ANC service at the University of Gondar Comprehensive Specialized Hospital ANC unit were the study population for the control group.

### Inclusion and exclusion criteria

Pregnant women with hypertension (blood pressure ≥ 140 / 90 mmHg) and proteinuria (urine protein > 1 + by urine dipstick) after 20^th^ GW [35], and attending the University of Gondar Comprehensive Specialized Hospital ANC unit during the data collection period were enrolled in this study as cases (PE). Age and GW matched NT pregnant women who attended the hospital for routine obstetric care, during the data collection period were used as control. 

Patients with a known history of hypertension, renal disease, liver disease, thyroid disease, diabetes mellitus, heart disease, thromboembolism or known thrombophilic disease, recurrent miscarriage, pre-term labor, intrauterine growth restriction, intrauterine fetal death, coagulation disorder, hematological malignancy and women with a recent major surgery or trauma, morbid obesity (body max index (BMI) ≥ 40 kg/m^2^), women with inflammatory diseases (asthma, allergy, rheumatoid arthritis, retinitis and patients with any sign of infection), anticoagulant treated women (aspirin, heparin, warfarin) and antihypertensive drug users were excluded from the study. The exclusion was accomplished by asking individuals directly if they had the conditions and/or reviewing their medical records using an exclusion criteria checklist.

### Sample size determination and sampling technique

Since the PE cases (study population) were small in the study period, the sample size was determined by using a census method [36]. Therefore, the entire PE cases who fulfilled the inclusion criteria within the study period were recruited by a convenient sampling technique. Moreover, age and GW matched NT pregnant women who attended the hospital for routine obstetric care during the data collection period were recruited as control by considering one to one ratio between cases (PE pregnant women) and controls (NT pregnant women).

### Data collection and laboratory procedures

#### Socio-demographic, clinical and obstetric data collection

Data related to socio-demographic characteristics including age, residence, educational status, marital status and occupation were collected by face-to-face interviews using a pretested questionnaire. The clinical and obstetrics data of both cases and controls were extracted from the patient’s chart (medical records) using a predesigned data collection format. Stadiometer (Infiniti Med Lab Pvt. Ltd., India) was used to measure the height of the participants. Participants stand erect on the floorboard of the Stadiometer with their backs to the vertical backboard. During the height measurement, the participant’s shoes and hats were removed. The height was measured to the nearest 0.1 cm without shoes and a hat [37]. The Weight of the participants was measured using a weight measurement scale (Infiniti Med Lab Pvt. Ltd., India). The weight scale was set to zero before starting the weight measurement. Participants were asked to remove extra layers of clothing, shoes, jewelry, and any items in their pockets then stand with their weight evenly distributed between both feet, arms hanging freely by the sides of the body, palms toward thighs and head up and facing straight ahead. Weights were finally recorded to the nearest 0.1 kg (100 gm) [37]. Then the BMI was calculated by dividing weight in (kg) by height squared in (m^2^) to screen the body fat ratio [37]. The patients were requested to sit upright with their upper arms positioned on the bench and participants’ BP was measured using an automatic digital sphygmomanometer (Omron Health Care Co., Ltd. Kyoto, Japan).

#### Laboratory sample collection and analysis

A total of six milliliters of blood was collected by syringe method using a 10 cc syringe with a 21 gauge needle at the antecubital vein immediately after the PE was diagnosed and before any intervention was taken. Three milliliters of the collected blood was transferred to a test tube containing ethylene diamine tetraacetic acid (EDTA) anticoagulant tube and adequately mixed with the anticoagulant by inverting the tube three to five times and used for complete blood count (CBC)*.* The remaining three milliliters of blood were transferred to a serum separator test tube for liver enzymes (Alanine transaminase (ALT), aspartate aminotransferase (AST)), total bilirubin and liver protein tests (total protein and albumin). A complete blood count (CBC) test was analyzed by five differential automated hematology analyzer SYSMEX XS-500i (Sysmex, Kobe, Japan) to determine leukocyte count with eosinophil count. Samples were examined within two hours after vein puncture. On the other hand, the liver enzymes, total bilirubin and liver proteins were examined using a Humastar 200 (Human GmbH, Germany) chemistry analyzer after the serum was separated from the cells by centrifugation for at least 5 min at 5000 revolutions per minute. All procedures were conducted according to the manufacturers’ instruction manual*.*

A random urine specimen was collected using a clean dry leak-proof urine cup. After labeling a leak-proof urine cup with the patient’s ID, the patient was instructed to fill half of the cup and bring it back. Then, proteinuria was determined by Cromatest® Linear URS-10 strip (Linear Chemicals S.L, 08,390 Montgat, and Barcelona, Spain) which is a semi-quantitative test. A dipstick is a thin plastic stick with chemical strips on it. The chemical strips change color if certain proteins are present or their levels are above normal. Small increases in protein in the urine usually aren’t a cause for concern, but larger amounts may indicate a kidney problem. A well-prepared data collection sheet was used to collect the entire laboratory-based data.

#### Data quality control

The questionnaire was first translated into the local language (Amharic) and then returned to English. The questionnaire was also pretested and training was given to the data collectors before the actual data collection. Close supervision of data collectors and review of the collected data for completeness and consistency were performed by the investigator of the study. Anthropometric and BP measures were performed twice, with the average being used.

All the sample collection procedures were carried out following standard operation procedures (SOP). The quality of the collected blood samples was checked for hemolysis, clot and correct volume. The performance of the instrument for the CBC test was monitored by background checking. Moreover, a morphologic examination was done as part of quality control. The chemistry analyzer was evaluated by running known pathological/abnormal and normal quality control materials for each analytical test, daily. The manufacturer’s instructions and SOP were strictly followed at each step of the laboratory analysis. The results were properly documented, transcribed and reviewed. Data were entered using the double-entry method to trace data entry errors which has a strong negative effect on study results and conclusions.

#### Data analysis and interpretation

The collected data was entered into Epi-data 4.6 software and then exported into a statistical package for social science statistical software version 20 (SPSS 20) (SPSS Inc., Chicago, IL, USA) for analysis. Data distribution was checked by the Shapiro–Wilk normality test. Comparison of normally distributed data between the two groups was done by independent t-test, and the results are expressed as mean ± standard deviation (SD), whereas the Mann–Whitney U test was used for comparison of non-normally distrusted data and the results are presented as median and Interquartile Range (IQR). After checking the significant differences in eosinophil count between PE and NT groups, receiver operative curve (ROC) analysis was performed to determine the area under the curve (AUC), sensitivity, and specificity for PE prediction. The Youden index was calculated to establish the optimal cut-off values of eosinophil count that showed the best combination of sensitivity and specificity for the diagnosis of PE. The binary logistic regression model was also used to identify factors associated with PE. The crude odds ratio (COR) and the adjusted odds ratio (AOR) were used to estimate the strength of the association for univariate and multivariate binary logistic regression, respectively. For all statistics**,** *P*-value < 0.05 was considered statistically significant.

## Results

### Socio-demographic and clinical characteristics of study participants

A total of 126 pregnant women were enrolled in this study (63 PE and 63 NT). Most of the study participants were urban residents (88; 69.8%) and 39 (31%) had no formal education. Moreover, most of them were housewives 46 (36.5%) followed by farmers 36 (28.6%). The patient’s ages ranged from 18 to 39 years old with a mean age of 27.8 ± 4.68 (28.1 ± 4.61 years old for PE and 27.5 ± 4.77 years old for NT) (Table 1).

**Table 1:** Socio-demographic characteristics of study participants attending the University of Gondar Comprehensive Specialized Hospital antenatal care unit, 2021 (*n* = 126)

**Variable type**	**Category**	**Study group**		**Total**	***P*-value**
		**Normotensive group**	**Preeclampsia group**		
Age: Mean ± SD		27.5 ± 4.77	28.1 ± 4.61	27.8 ± 4.68	0.449
Residence: N (%)	Urban	46 (73)	42 (66.7)	88 (69.8)	0.437
	Rural	17 (27)	21 (33.3)	38 (30.2)	
Educational status: N (%)	No formal education	19 (30.2)	20 (31.7)	39 (31.0)	0.956
	Primary	12 (19)	11 (17.5)	23 (18.3)	
	Secondary	19 (30.2)	17 (27)	36 (28.6)	
	Diploma and above	13 (20.6)	15 (23.8)	28 (22.2)	
Marital status: N (%)	Married	59 (93.7)	62 (98.4)	121 (96)	0.171
	Single	4 (6.3)	1 (1.6)	5 (4)	
Occupation: N (%)	House wife	26 (41.3)	20 (31.7)	46 (36.5)	0.703
	Farmer	16 (25.4)	20 (31.7)	36 (28.6)	
	*Government* employee	11 (17.5)	11 (17.5)	22 (17.5)	
	Private	10 (15.9)	12 (19)	22 (17.5)	

*P*-value by Pearson chi-square for categorical variable

There were no significant variations in GWs, gravidity and parity between the control and PE groups. However, SBP and DBP of PE patients were significantly higher compared to the control groups with median (IQR) of 140 mmHg (140–150) and 90 mmHg (90–100) in the PE patients, and 100 mmHg (100–110) and 73.3 mmHg (60–80) in the NT group respectively. On the other hand, BMI was found to be lower in PE pregnant women than in NT pregnant women; Median (IQR) (21.4 kg/m^2^ (20.5–22.7) vs. 23.0 kg/m^2^ (20.7–24.9)) (*p* < 0.05) (Table 2).

**Table 2:** Clinical and obstetric characteristics of study participants attending the University of Gondar Comprehensive Specialized Hospital antenatal care unit, 2021 (*n* = 126)

**Variable**	**Category**	**Study group**		***P*-value**
		**Normotensive group (*n* = 63)**	**Preeclampsia group (*n* = 63)**	
Gravidity n (%)	Primagravida	21(33.3)	23 (36.5)	0.852^a^
	Multigravida	42 (66.7)	40 (63.5)	
Parity n (%)	Nulliparous	26 (41.3)	21(33.3)	0.177^a^
	Primiparous	20 (31.7)	15 (23.8)	
	Multiparous	17 (27.0)	27 (42.9)	
GW	Median (IQR)	33 (30–38)	35 (31–38)	0.850^d^
BMI (kg/m^2^)	Median (IQR)	23 (20.7–24.9)	21.5 (20.5–22.7)	0.020^d^
SBP (mmHg)	Median (IQR)	100 (100–110)	140 (140–150)	< 0.001^d^
DBP (mmHg)	Median (IQR)	70 (60–80)	90 (90–100)	< 0.001^d^
MAP (mmHg)	Median (IQR)	80 (73.3–86.7)	110 (106.7–116.7)	< 0.001^d^

^a^ Pearson chi-square and

^d^ Mann–Whitney U independent t-test statistics

### Leukocyte parameters and liver function tests among study groups

According to the current study, the median total leukocyte and the differential count (both absolute and relative) of neutrophils, lymphocytes, monocytes and basophils were not statistically significant between PE patients and NT pregnant women (U statistics test; *p* > 0.05). However, the median eosinophil cell count (both absolute and relative) of PE pregnant women significantly lower than the NT pregnant women [absolute count: median (IQR); 50 Cells/µL (10 -200) vs. 120 Cells/µL (60 – 270); (U statistics test; *p* = 0.002) and relative count: median (IQR); 0.7% (0.2 – 2.4) vs. 1.3% (0.8 – 3.0); (U statistics test; *p* = 0.019)]. Most of the PE patients were Eosinopenia and it was statistically different from the NT pregnant women (50.8% vs. 22.2%; Fisher’s exact test; *p* = 0.001). The liver function tests results; AST, ALT and total bilirubin were also significantly higher in the PE pregnant women than in NT pregnant women. But, total protein and albumin had no significant difference between NT and PE pregnant women (*p*-value > 0.05) (Table 3).

**Table 3:** Comparisons of proteinuria, liver tests and total leukocyte and differential count between preeclamptic and healthy pregnant women attending the University of Gondar Comprehensive Specialized Hospital antenatal care unit, 2021 (*n* = 126)

**Variable**	**Category**	**Study group**		***P*-value**	**Normal**^c^** value**
		**Normotensive group (*n* = 63)**	**Preeclampsia group (*n* = 63)**		
Proteinuria n (%)	Positive	2 (3.2)	63 (100)	< 0.001^a^	Negative
	Negative	61(96.8)	0 (0)		
Total leukocyte	Cells/µL: Median (IQR)	7180 (6020—9280)	6990 (5290 – 9450)	0.405^b^	3200 – 8800
Neutrophil	Cells/µL: Median (IQR)	5090 (3840—6810)	5150 (3230—7020)	.581^b^	1500 – 7000
	%: Median (IQR)	68.5 (63.3—73.2)	67.3 (61.4—79.4)	0.678^b^	40 – 70
Lymphocyte	Cells/µL: Median (IQR)	1570 (1370 -1890)	1540 (1110—1890)	0.156^b^	1000 – 3700
	%: Median (IQR)	22 (17.8—26.2)	22.1 (14.6—28.7)	0.724^b^	20 – 50
Monocytes	Cells/µL: Median (IQR)	530 (410 – 670)	460 (350—620)	0.064^b^	130 – 700
	%: Median (IQR)	7 (5.8—8.4)	6.7 (5.6—7.6)	0.32^b^	4 – 8
Basophile	Cells/µL: Median (IQR)	20 (10—20)	20 (10—20)	0.823^b^	0 – 100
	%: Median (IQR)	0.2 (0.1—0.3)	0.2 (0.1—0.3)	0.703^b^	0 – 2
Eosinophil	Cells/µL: Median (IQR)	120 (60—270)	50 (10—200)	**0.002**^**b**^	0 – 400
	%: Median (IQR)	1.3 (0.8—3.0)	0.7 (0.2—2.4)	**0.019**^**b**^	0 – 6
	Eosinophilia n (%)	3 (4.8)	0 (0)	**0.001**^**a**^	-
	Normal count n (%)	4 (73.0)	31 (49.2)		
	Eosinopenia n (%)	14 (22.2)	32 (50.8)		
AST	U/L:Median (IQR)	22 (19–26)	41 (25–73)	** < 0.001**^**b**^	0 – 31
ALT	U/L:Median (IQR)	13 (10–16)	24 (15–41)	** < 0.001**^**b**^	0 – 32
Total bilirubin	mg/dL:Median (IQR)	0.5 (0.37–0.67)	0.61 (0.44–0.8)	**0.02**^**b**^	0.1 – 1.2
Albumin	g/dL:Median (IQR)	3.18 (2.69–3.49)	3.11 (2.74–3.34)	0.215^b^	3.8–5.1
Total protein	g/dL:Median (IQR)	5.88 (4.68–6.5)	5.68 (4.59–6.1)	0.115^b^	6.6–8.7

^a^ Fisher’s exact test

^b^ Mann–Whitney U statistics test

^c^ The normal values were taken from the University of Gondar Comprehensive Specialized Hospital Laboratory where the test was analyzed

### Leukocyte parameter and liver function test between early-onset and late-onset preeclampsia

Among the PE pregnant women, 30 (47.6%) had early-onset PE (PE diagnosed before 34 weeks of gestation) and the rest had late-onset PE (PE diagnosed after 34 weeks of gestation). The comparison of early-onset and late-onset PE showed that there were no statistically significant differences in terms of total and differential count of the leukocyte, including the eosinophil count (*P*-value > 0.05). Moreover, the liver enzyme tests (AST and ALT), total protein and albumin were not showed a statistically significant difference between early-onset and late-onset PE (*P*-value > 0.05). But, there was a statistically significant difference between early-onset and late-onset PE in terms of total bilirubin (*P*-value = 0.027 (Table 4).

**Table 4:** Comparisons of complete blood cell count and liver function test results between early-onset and late-onset preeclamptic pregnant women attending the University of Gondar Comprehensive Specialized Hospital antenatal care unit, 2021 (*n* = 63)

**Variable**	**Category**	**Preeclampsia group**		***P*-value**
		**Early-onset (*n* = 30)**	**Late-onset (*n* = 33)**	
Leukocyte count	Cells/µL: Median (IQR)	6955 (5230—10,002)	7600 (5220 – 9095)	0.254^b^
Neutrophil	Cells/µL: Median (IQR)	4650 (3100—7073)	5260 (3405—7035)	.895^b^
	%: Median (IQR)	67.2 (57.9 – 75.9)	67.6 (62.4 – 80.3)	0. 895^b^
Lymphocyte	Cells/µL: Median (IQR)	1575 (1215 -2235)	1360 (1105—1840)	0.263^b^
	%: Median (IQR)	22.2 (16.5 – 34.5)	20.3 (13.4 – 26.9)	0.895^b^
Monocytes	Cells/µL: Median (IQR)	455 (383 – 643)	470 (315—585)	0.895^b^
	%: Median (IQR)	6.7 (6.0 – 7.5)	6.8 (3.8—7.7)	0.895^b^
Basophile	Cells/µL: Median (IQR)	20 (10—20)	10 (10—20)	0.815^b^
	%: Median (IQR)	0.2 (0.1—0.3)	0.2 (0.1—0.3)	0.876^b^
Eosinophil	Cells/µL: Median (IQR)	55 (20—205)	50 (10—205)	0.895^b^
	%: Median (IQR)	0.75 (0.3 – 2.3)	0.6 (0.1—3.4)	0.914^b^
	Normal count n (%)	11 (36.7)	14 (42.4)	0.797^b^
	Eosinopenia n (%)	19 (63.3)	19 (57.6)	
AST	U/L:Median (IQR)	41 (26.8 – 76.3)	32 (24–53)	0.322^b^
ALT	U/L:Median (IQR)	23.5 (16.8—41)	24 (14–37.5)	0.469^b^
Total bilirubin	mg/dL:Median (IQR)	0.7 (0.52 -0.84)	0.56 (0.39 -0.70)	**0.027**^**b**^
Albumin	g/dL: Mean ± 2SD	3.07 ± 1.2	3.04 ± 0.75	0.786^c^
Total protein	g/dL: Mean ± 2SD	5.40 ± 2.2	5.60 ± 3.4	0.525^c^

^b^ Mann–Whitney U statistics test

^c^ independent t-test

### Factors associated with preeclampsia

The proportion of PE was higher in higher age groups (56.4% vs. 45.1%). It was also higher among pregnant women with BMI lower than 25 kg/m^2^ (54.3% vs. 28.6%), pregnant women who had Eosinophil count ≤ 55 cells/µL *(*69.6% vs. 38.7%) and pregnant women with multigravida (51.2% vs. 47.7%). The result also showed that the proportion of PE was higher among Multiparous pregnant women (61.4%) followed by nulliparous (44.7%). The current result was also showed that PE was higher in patients with abnormal liver enzyme tests (57.1% vs. 7.9% for AST and 30.2% vs.1.6% for ALT).

However, in the bivariable binary logistic regression analysis, PE was associated only with BMI < 25 kg/m^2^ (COR = 2.97; 95% CI: 1.07–8.25), eosinophil count ≤ 55 cells/µl (COR = 3.76; 95% CI: 1.53 – 9.22) and AST (COR = 15.47**;** 95% CI: 5.46 – 43.8**)**. Moreover, in the multivariable model, controlling the confounding factor, only eosinophil count ≤ 55 cells/µl (AOR = 3.56; 95% CI: 1.62 – 7.80) and AST (AOR = 14.86; 95% CI: 4.97—44.4**)** were significantly associated with the development of PE. ALT was not entered inter to multivariable model, because it did not fulfill the binary logistic regression assumption; but it had a statistically significant association with PE (fisher exact test *p*-value < 0.001) (Table 5).

**Table 5:** Preeclampsia and associated factors among pregnant women attending the University of Gondar Comprehensive Specialized Hospital antenatal care unit, 2021 (*n* = 126)

Variable	Category	Group		COR (95% CI)	AOR (95% CI)	*P* Value
		Preeclampsia n (%)	Normotensive n (%)			
Age in years	18 – 28	32 (45.1)	39 (54.9)	1	1	0.633^a^
	> 28	31 (56.4)	24 (43.6)	1.57 (0.78 – 3.2)	0.78 (0.28 – 2.15)	
BMI in kg/m^2^	< 25	57 (54.3)	48 (45.7)	**2.97 (1.07 – 8.25)**	1.42 (0.46 – 4.22)	0.548^a^
	≥ 25	6 (28.6)	15 (71.4)	1	1	
Eosinophil count cells/µL	≤ 55	32 (69.6)	14 (30.4)	**3.61 (1.67 – 7.82)**	**3.76 (1.53 – 9.22)**	0.004^a^
	> 55	31 (38.7)	49 (61.3)	1	1	
Gravidity	Primagravida	21 (47.7)	23 (52.3)	1	-	0.709^b^
	Multigravida	42 (51.2)	40 (48.8)	1.15 (0.55 – 2.39)	-	
Parity	Nulliparous	21(44.7)	26 (55.3)	1	1	-
	Primiparous	15 (42.9)	20 (57.1)	0.93 (0.38 – 2.24)	0.99 (0.38 – 2.58)	0.983^a^
	Multiparous	27 (61.4)	17 (38.6)	1.97 (0.85 – 4.54)	2.23 (0.91 – 5.46)	0.078^a^
AST	Normal	27 (42.9)	58 (92.1)	1		< 0.001^a^
	High	36 (57.1)	5 (7.9)	**15.47 (5.46 – 43.8)**	**14.86 (4.97—44.4)**	
ALT	Normal	44 (69.8)	62 (98.4)	**-**	**-**	< 0.001^c^
	High	19 (30.2)	1(1.6)	**-**	**-**	

*COR* Crude odds ratio, *AOR* Adjusted odds ratio

^a^ adjusted odds ratio *p*-value

^b^ crude odds ratio *p*-value

^c^ Fischer exact test *p*-value

### Diagnostic values of eosinophils

According to the ROC curve analysis, the eosinophil absolute count cut-off value ≤ 55 cells/µL had an AUC of 0.66 (95% CI; 0.56—0.75) with a sensitivity of 50.8% and specificity of 77.8%. This cut-off value also had a positive predictive value (PPV) and negative predictive value (NPV) of 69.6% and 61.3%, respectively. On the other hand, the eosinophil relative count ≤ 0.75% had an AUC of 0.62 (95% CI; 0.52–0.72) which had sensitivity and specificity of 52.4% and 76.2%, respectively. The PPV and NPV were 68.8% and 61.5%, respectively (Table 6).

**Table 6:** The diagnostic values of eosinophil count for the prediction of PE among pregnant women attending the University of Gondar Comprehensive Specialized Hospital ANC unit, 2021 (*n* = 126)

**Eosinophil parameter**	**AUC (95% CI)**	**Cut-off value**	**Sensitivity (%)**	**Specificity (%)**	**PPV**	**NPV**
Absolute count	0.66 (0.56—0.75)	≤ 55 cells/µL	50.8%	77.8%	69.6	61.3
Relative count	0.62 (0.52–0.72)	≤ 0.75%	52.4%	76.2%	68.8	61.5

## Discussion

Preeclampsia is a pregnancy-related clinical syndrome that is associated with increased systemic vascular resistance, endothelial cell dysfunction, and hematological abnormalities [38]. Measurement of these abnormal Parameters is a valuable marker for the prediction of PE [39, 40]. This study is a comparative cross-sectional study that aimed to compare eosinophil count among PE and NT pregnant women and to determine the diagnostic values of eosinophil count for the prediction of PE.

According to the current study, eosinophil count was significantly lower in PE groups compared to NT pregnant women (absolute eosinophil count median (IQR): 50 cells/µL (10 -200) vs. 120 cells/µL (60 -270); Mann–Whitney U test statistics *P*-value = 0.002). This study also revealed that Eosinopenia, defined as ≤ 55cells/µL, was more prevalent in PE groups than in NT groups (50.8% vs. 22.2%) and it was statistically significant (Pearson chi-Squared *P*-value = 0.001). The reason might be due to increased direct eosinophil apoptosis induced by type 1 interferons. Type 1 interferons were important molecules in eosinophil cell apoptosis [22] and their gene expression was increased in preeclamptic women [25]. Interleukin-5 is a crucial cytokine for the differentiation and survival of eosinophil cells [14] and the secretion of interleukin-5 in PE pregnant women was significantly lower than in NT pregnant women [26]. Therefore, the other possible reason might be due to a decrease in the differentiation of eosinophils from the bone marrow and a reduction in survival of circulating Eosinophils due to low IL-5 in the blood circulation of PE groups. It is also known that PE is more associated with a maladaptive immune response of cells and a hyper-inflammatory state [3, 41]. In inflammation, mediators released from epithelial cells or inflammatory cells induce the migration of eosinophils from the blood into the affected tissues [21]. Hence, without bone marrow compensation, the migration of eosinophils from blood circulation might reduce the eosinophil count in peripheral blood. The other known cause of eosinopenia is stress and mediated by adrenal glucocorticosteroids and epinephrine [27]. Stress induces the release of adrenal Glucocorticoids and/or epinephrine. Glucocorticoids are immune-suppressors that decline the cytokines such as eotaxins and inhibition of the cytokine-dependent survival of eosinophils. Glucocorticoids also suppress the transcription of a number of genes involved in eosinophil production and trafficking, including IL-3, IL-4, IL-5, and GM-CSF [42]. According to Zhang et.al, Meta-analysis, Stress was increased by 49% in PE pregnant women compared to NT pregnant women [28]. So, this might be the other possible cause of eosinopenia in PE pregnant women.

The current finding showed that Eosinopenia had clinical significance in the diagnosis of preeclamptic pregnant women. In other words, the odds of being PE was 3.56 times higher among pregnant women who had eosinophil count ≤ 55 cells/µL than pregnant women who had eosinophil count > 55 cells/µL (AOR = 3.76; 95% CI: 1.53 – 9.22). Similar studies were found by Lurie et.al [5] and Mtali et.al [8]. The clinical significance of Eosinopenia was also reported as a reliable marker of sepsis [24, 43], infection [16, 44], COVID-19 patients [22, 23] and prognostic factor in the exacerbation of chronic obstructive pulmonary disease [45].

The receiver operating characteristic curve analysis of the current study showed that eosinopenia at a cut-off value ≤ 55cells/µL was reasonable in the diagnosis of PE (AUC (95% CI):0.66 (0.56 – 0.75). It had a sensitivity of 50.8% and specificity of 77.8% with PPV and NPV of 69.6% and 61.3%, respectively. This was comparable with the diagnostic value of platelet count (AUC: 0.624—0.653) [3, 46]. Moreover, Compare to the Nooh et.al study, the current study showed that eosinophil count had a better diagnostic value than platelet count [47]. It was also comparable with the diagnosis value of mean platelet volume (AUC: 0.638) [3] and platelet distribution width (AUC: 0.621- 0.742) [3, 11]. However, some studies showed the diagnostic value of mean platelet volume and platelet distribution width were better than the current finding ( AUC: 0.78- 0.94) [11, 47] and (AUC: 0.98) [47], respectively.

The current study also revealed that eosinopenia had also a comparable diagnostic value of NLR and platelet to lymphocyte ratio (PLR) [9]. The current finding also revealed that eosinophil count had a better diagnostic value than NLR and PLR. Toptas et al. demonstrated that NLR and PLR were not significantly different between the pregnant women with PE and NT pregnancies (had no diagnostic value) [2]. On the other hand, Agrawal et.al showed that NLR and PLR had better diagnostic values than the current finding, eosinopenia (AUC: 0.80 for both parameters) [48]. The difference might be due to that the ratio can be affected by the statistical significance of the parameter that is used to calculate the ratio. For a brief description, in the study conducted by Agrawal et.al the neutrophil and platelet counts were not significantly different between PE and NT pregnant women, but, the lymphocyte count was significantly different. So, the NLR and PLR showed a significant difference due to the lymphocyte count difference [48]. The role of eosinopenia as a diagnostic value was also reported in COVID-19 infection (AUC: 0.97) [22] and sepsis (AUC: 0.84) [49].

The current study also tried to assess the association between liver function tests and PE. The result showed that PE was significantly associated with AST and ALT. The odds of PE were nearly 15 (95% CI: 4.97—44.4**)** times more likely among pregnant women with abnormal AST results compare to the pregnant women with normal AST results. In addition, PE was higher among pregnant women with abnormal ALT results compare to pregnant women with normal ALT results. In other words, ALT abnormality was more common among preeclampsic pregnant women than NT pregnant women (30.2% vs. 1.6%; Fischer exact test *p*-value < 0.001). Other studies also revealed that AST and ALT were significantly increased in PE [50, 51]. Elevated serum AST and ALT in preeclampsia are due to the effect of hypoxia on the liver. In PE, endothelial cells are destroyed, resulting in decreased Prostacyclin levels and increased Thromboxane levels. This causes vasoconstriction in the liver and reduces blood flow and leads to liver hypoxia. Due to the effects of hepatic hypoxia, hepatocytes undergo necrosis and degeneration, thus increasing AST and ALT levels [52].

### Strengths and limitations of the study

To our best knowledge, this study was the first finding in the prediction of PE by using eosinophil count. This study also provides baseline information for father studies. However, the study had limitations. One of the limitations was being a single-center study, which limits the generalizability of the findings to the local community. Another drawback of this study is that the sample size was relatively small, which may limit the statistical power of the study. Additionally, because the study was a cross-sectional study the data were limited to show the dynamic change of eosinophil count on the course of Pregnancy. The study participants were selected by a convenient technique that might introduce selection bias. Furthermore, exclusion was made by asking individuals directly if they had the conditions and/or reviewing their medical records for the presence of conditions, which might be affected by remembering bias. Additionally, parasite infections were not assessed both for PE and NT.

## Conclusions and recommendations

Based on the current findings, eosinophil count was significantly decreased in PE groups. The eosinophils count ≤ 55cells/µl had reasonable/acceptable AUC in the diagnosis of PE. In other words, the odds of being PE were significantly higher in pregnant women who had eosinophil count ≤ 55 cells/µl. In addition, the liver function tests; AST and ALT were significantly associated with PE; therefore, important in the diagnosis of PE. However, the current study should be interpreted considering the following limitations. On one hand, the sample size was relatively small. On the other hand, it was a single-center and cross-sectional study. Additionally, there might be recalling bias on medical conditions like allergy, asthma and other chronic disease and parasite infection were not assessed both for PE and NT. Therefore; multicenter longitudinal studies with a large sample size are recommended to verify the role of eosinophil count in the diagnosis of PE and to evaluate their role at various GW of pregnancy.

## Data Availability

The datasets used and/or analyzed during the current study are available from the corresponding author and can access on reasonable request.

## Abbreviations

ALT: Alanine Transaminase
ANC: Antenatal Care
AOR: Adjusted Odds Ratio
AST: Aspartate aminotransferase
AUR: Area Under Curve
BMI: Body Max Index
CBC: Complete Blood Count
CI: Confidence Interval
COR: Crude Odds Ratio
EDTA: Ethylene Diamine Tetra acetic Acid
GW: Gestational Week
IQR: Inter Quartile Range
NLR: Neutrophil Lymphocyte Ratio
NT: Normotensive
NPV: Negative Predictive Value
PE: Preeclampsia
PLR: Platelet Lymphocyte Ratio
PPV: Positive Predictive Value
SD: Standard Deviation
SOP: Standard Operation Procedure
